# Association Between Dietary Calcium or Dairy Product Intake and Metabolic Syndrome Risk: A Systematic Review and Meta-Analysis

**DOI:** 10.3390/nu18061006

**Published:** 2026-03-22

**Authors:** Stefano Gonnelli, Antonella Al Refaie, Sara Gonnelli, Caterina Mondillo, Guido Cavati, Alessandra Cartocci, Carla Caffarelli

**Affiliations:** Department of Medicine, Surgery and Neuroscience, University of Siena, 53100 Siena, Italy; antonellaalrefaie@gmail.com (A.A.R.); saragonnelli@libero.it (S.G.); caterinamondillo28@gmail.com (C.M.); guido.cavati@student.unisi.it (G.C.); carla.caffarelli@unisi.it (C.C.)

**Keywords:** metabolic syndrome, dietary calcium intake, dairy products, meta-analysis

## Abstract

**Background**: Dietary calcium and dairy products are hypothesized protective factors against metabolic syndrome (MetS), yet epidemiological evidence remains inconsistent. This systematic review and meta-analysis evaluated the association between total dietary calcium intake or dairy consumption and MetS prevalence in adults. **Methods**: Following PRISMA 2020 guidelines, PubMed, Cochrane Library, ClinicalTrials.gov, and SCOPUS were searched through to October 2025 for eligible cross-sectional studies assessing dietary calcium or dairy intake and MetS (NCEP ATP III, IDF, or JIS criteria). Longitudinal studies, non-English articles, and pediatric populations were excluded. Quality was assessed via an adapted Newcastle–Ottawa Scale. Random-effects meta-analyses pooled fully adjusted odds ratios (ORs) and 95% confidence intervals (CIs) comparing the highest versus lowest intake categories. **Results**: Twenty-four studies were included (12 for dietary calcium intake, 12 for dairy products). Higher dietary calcium intake was significantly associated with lower MetS odds (pooled OR: 0.85; 95% CI: 0.80–0.91), despite substantial heterogeneity (I^2^ = 70.1%). Higher dairy consumption was also inversely associated with MetS (pooled OR: 0.78; 95% CI: 0.72–0.85; I^2^ = 64.6%). While small-study effects were observed for dairy, trim-and-fill analysis confirmed the robustness of the findings. Higher calcium intake further correlated with favorable profiles in individual MetS components, including blood pressure, HDL cholesterol, waist circumference, triglycerides, and fasting glucose. **Conclusions**: Higher total dietary calcium intake and dairy product consumption are associated with a lower prevalence of MetS in adults. However, the cross-sectional nature of the included studies precludes any inference of causality between calcium intake and MetS. Therefore, although these findings suggest a protective role of calcium-rich diets, well-designed prospective and interventional studies are warranted to clarify whether this relationship is causal.

## 1. Introduction

Metabolic syndrome (MetS), first described by Reaven in 1988 as “Syndrome X,” is characterized by the clustering of metabolic risk factors that substantially increase the risk of cardiovascular disease and type 2 diabetes mellitus [1]. Diagnostic criteria included the presence of at least three of the following components: central obesity, arterial hypertension, fasting hyperglycemia, hypertriglyceridemia, and low HDL cholesterol levels [2]. MetS reflects a complex metabolic dysfunction, frequently associated with insulin resistance and chronic low-grade inflammation, and represents a major determinant of cardiovascular morbidity and mortality [3]. The prevalence of MetS is increasing worldwide, particularly in Western countries, the Americas, and the Eastern Mediterranean region, driven by sedentary lifestyles, rising obesity rates, and population aging [4]. In Western populations, MetS affects approximately 20–35% of adults; in the United States, about 34% of adults meet ATP III criteria, while Europe shows comparable prevalence with notable regional variability [4,5]. In Italy, epidemiological studies report prevalence rates between 25% and 30%, with higher rates among older individuals, men, and those with obesity, and a narrowing of sex differences after menopause [6].

Despite extensive research, the pathogenesis of MetS remains only partially understood. Both genetic and environmental factors contribute, with diet playing a central role. Diets rich in simple sugars and saturated or trans fats and poor in fiber and micronutrients promote insulin resistance, inflammation, and endothelial dysfunction [7]. Conversely, healthy dietary patterns such as the Mediterranean and Dietary Approaches to Stop Hypertension (DASH) diets have been shown to improve glucose and lipid metabolism and favorably influence individual MetS components, including waist circumference, triglycerides, HDL cholesterol, and fasting glucose [7,8,9,10].

Growing epidemiological evidence suggests that higher consumption of dairy products—particularly low-fat and fermented types—is associated with a lower risk of MetS and improvements in its individual components [11,12,13]. Although dairy foods contain multiple nutrients that may affect metabolic health, calcium has been proposed as a key contributor. Observational studies have consistently reported inverse associations between dietary calcium intake and MetS prevalence, particularly in women [14,15]. Higher calcium intake has been linked to reduced obesity risk, possibly through effects on lipogenesis and fat oxidation [16], as well as improvements in insulin secretion, blood pressure regulation, and lipid metabolism [17,18]. However, other studies have failed to confirm these associations, yielding inconsistent findings [19,20]. In summary, while evidence suggests a potential link between dietary calcium intake and MetS, current data remain inconclusive.

Therefore, this systematic review and dose–response meta-analysis aims to examine evidence from cross-sectional studies on the association between dietary calcium intake or dairy products and metabolic syndrome, to better clarify the nature of this relationship.

## 2. Materials and Methods

### 2.1. Search and Assessment of Studies

**Figure 1:**
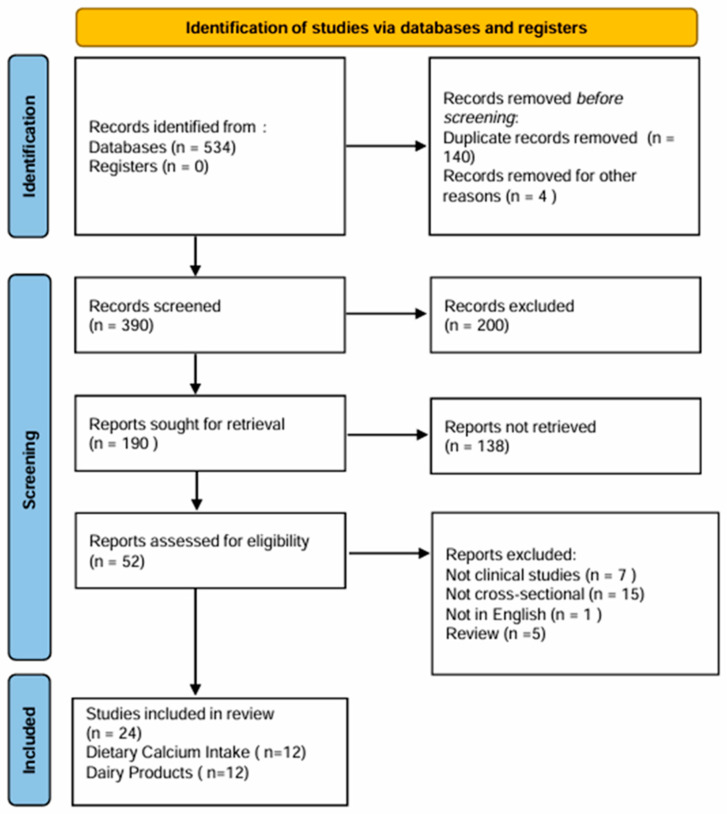
PRISMA flow diagram illustrating the study selection process for inclusion and exclusion in the systematic review and meta-analysis.

For this review we considered all relevant articles published up to October 2025. The PubMed/Medline, Cochrane Library, ClinicalTrials.gov, and SCOPUS databases were searched using the following search terms: “calcium intake” or “dietary calcium”, “dairy foods/products consumption” and “metabolic syndrome” or “MetS”. The process of selecting the studies for review in adherence to the PRISMA 2020 (Preferred Reporting Items for Systematic reviews and Meta Analyses) [21] is shown in Figure 1. Moreover, the protocol was registered in PROSPERO with code CRD420261328992. After excluding irrelevant articles, duplicates, case–control, cohort, reviews, and meta-analyses, 24 studies were deemed eligible for inclusion in the review. Of these, 12 examined the relationship between total dietary calcium intake and metabolic syndrome, while 12 investigated the association between dairy products consumption and metabolic syndrome, thereby forming two distinct groups that were analyzed separately. All included studies had a cross-sectional design. In all cases, daily dairy food consumption and total dietary calcium intake were assessed using dietary questionnaires. The exclusion criteria were as follows: (i) cohort and longitudinal studies; (ii) studies written in languages other than English; (iii) studies assessing the association between dietary calcium intake or dairy consumption and one or only some components of metabolic syndrome, rather than metabolic syndrome as a whole; and (iv) studies including participants under 18 years of age or with specific pathologies.

### 2.2. Methodological Quality and Risk of Bias of Studies

Methodological quality and risk of bias of the included cross-sectional studies were assessed using an adapted version of the Newcastle–Ottawa Scale (NOS). This tool evaluates studies across three domains: Selection (maximum 4 stars), Comparability (maximum 2 stars), and Outcome (maximum 3 stars), with a total possible score of 9 stars. Overall study quality was categorized as low (0–4 stars), moderate (5–6 stars), or high (7–9 stars). Two investigators (A.A. and G.S.) independently reviewed all identified studies and performed quality assessment; any disagreements regarding study eligibility were resolved through discussion and consensus (Table S1A,B of the Supplementary Material).

In all the selected studies, metabolic syndrome was defined exclusively according to one of the following criteria: (1) the Third Report of the National Cholesterol Education Program Adult Treatment Panel (NCEP ATP III) [22], (2) the International Diabetes Federation definition (IDF) [23], or (3) the Joint Interim Statement of the International Diabetes Federation (JIS) [24].

To assess the certainty of evidence, we applied the GRADE (Grading of Recommendations, Assessment, Development, and Evaluations) approach, separately evaluating the studies that considered dietary calcium intake and those that assessed dairy product consumption (Table S2A,B of the Supplementary Material) [25].

### 2.3. Statistical Analysis

Two separate random-effects meta-analyses were conducted to calculate the overall association between the prevalence of metabolic syndrome in studies that evaluated total dietary calcium intake and in those that evaluated dairy product intake, respectively. For each study, the most fully adjusted odds ratio (ORs) and 95% confidence intervals (CIs) comparing the highest versus the lowest categories of intake were extracted and pooled. Random-effects models were applied to account for between-study variability. Statistical heterogeneity was assessed using Cochran’s Q-test and quantified with the I^2^ statistic, calculated as

$$I^{2}=100\%\times \frac{\left(Q-df\right)}{Q}$$

where Q represents Cochran’s heterogeneity statistic and df the degrees of freedom. I^2^ values of <50%, 50–75%, and >75% were considered indicative of low, moderate, and high heterogeneity, respectively. A Q-test *p*-value < 0.05 was considered statistically significant. The between-study variance (τ^2^) was estimated within the random-effects framework. Potential publication bias was evaluated by visual inspection of Begg’s funnel plots and statistically assessed using Begg’s rank correlation test and Egger’s regression test. A *p*-value < 0.05 was considered statistically significant. The Greenland and Longnecker method was used to evaluate the dose response meta-analysis [26].

## 3. Results

According to our search strategy, twenty-four cross-sectional articles were selected and included in this review. Of these, 12 articles focused on the association between total dietary calcium intake and metabolic syndrome, while the remaining 12 explored the possible association between daily consumption of dairy foods and metabolic syndrome.

### 3.1. Total Dietary Calcium Intake and Metabolic Syndrome

Table 1 presents the main characteristics of the eligible cross-sectional studies included in the systematic review and meta-analysis examining the association between dietary calcium intake and metabolic syndrome in adults [19,20,27,28,29,30,31,32,33,34,35,36]. Overall, the included studies comprised a total of 73,652 men and 128,058 women. With regard to study location, eight studies were conducted in Asia, two in the United States, one in Brazil, and one in Australia. Dietary calcium intake was assessed using food frequency questionnaires (FFQs) and/or 24–48 h dietary recalls. Study quality was rated as high in eight studies, moderate in three, and low in one [Table S1A in Supplementary Materials].

**Figure 2:**
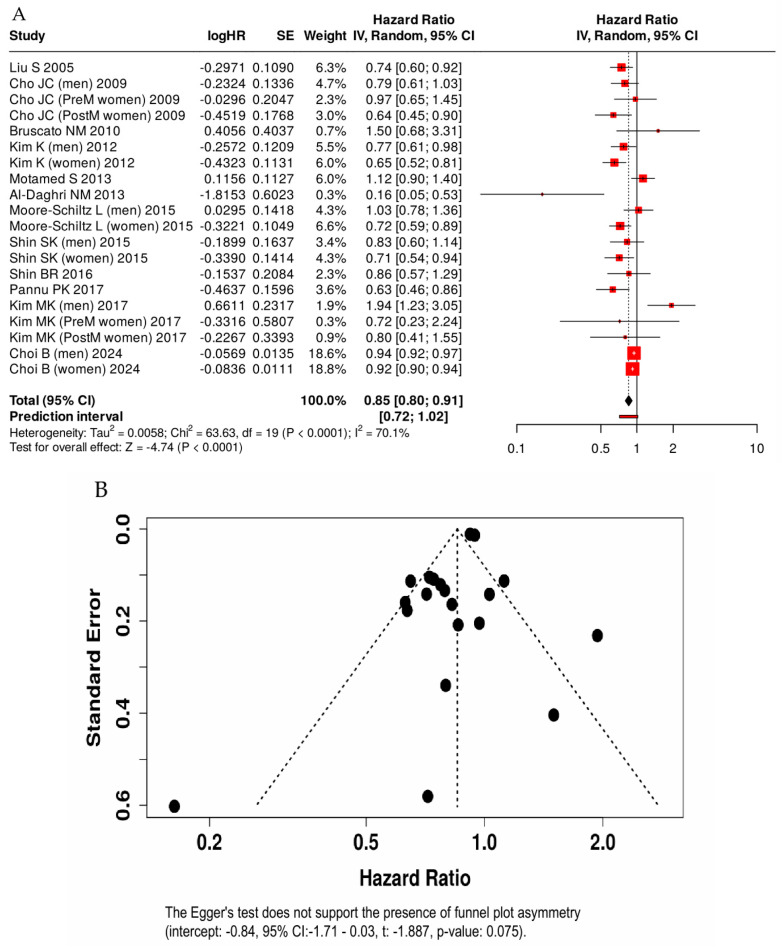
Forest plot (**A**) and funnel plot (**B**) of the association between the highest versus lowest category of dietary calcium intake and metabolic syndrome in adults [19,20,27,28,29,30,31,32,33,34,35,36].

Moreover, all studies were conducted in apparently healthy participants. Across studies, higher dietary calcium intake was associated with lower odds of metabolic syndrome, with a pooled odds ratio of 0.85 (95% CI: 0.80–0.91) [Figure 2]. Thus, the highest level of dietary calcium intake, compared with the lowest, was associated with a statistically significant 15% reduction in the odds of metabolic syndrome. Substantial relative heterogeneity was observed (I^2^ = 70.1%); however, the estimated between-study variance was small (τ^2^ = 0.005; τ ≈ 0.07), indicating limited absolute variability in effect sizes on the log-odds scale. Visual inspection of the funnel plot of log (OR) versus standard error did not indicate substantial asymmetry. This observation was supported by Egger’s regression test (*p* = 0.075) and Begg’s test (Kendall’s τ = −0.02, *p* = 0.92), suggesting no strong evidence of publication bias among the included studies.

**Figure 3:**
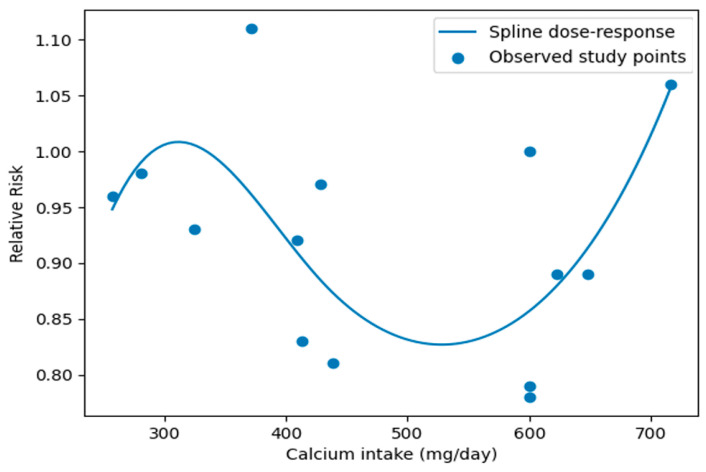
Nonlinear dose–response spline: dairy calcium intake and metabolic syndrome.

Meta-regression analyses were conducted to explore potential sources of heterogeneity, including geographic region, sex group, and dietary assessment method. The analysis showed that sex significantly moderated the association between dietary calcium intake and metabolic syndrome (β = −0.275, *p* = 0.038), indicating an inverse association in women compared with men. Geographic region and dietary assessment method were not significantly associated with Mets. Dose–response relationships were examined using a two-stage random-effects dose–response meta-analysis based on the method of Greenland and Longnecker. A linear dose–response analysis including 10 studies showed that each 100 mg/day increment in dietary calcium intake was associated with an approximately 2% lower odds of metabolic syndrome (OR = 0.98). Accordingly, a 300 mg/day increment corresponded to an estimated 6% lower odds of MetS (OR = 0.94) (Table S3A of the Supplementary Material). Potential nonlinear dose–response relationships were evaluated using restricted cubic splines with three knots placed at the 10th, 50th, and 90th percentiles of the exposure distribution. The nonlinear analysis indicated a significant association between dietary calcium intake and MetS, with the greatest reduction in MetS odds observed at approximately 500 mg/day of dietary calcium intake. Beyond this level, further increases in calcium intake were not associated with additional reductions in MetS odds [Figure 3]. This pattern suggests a potential threshold effect, whereby moderate dietary calcium intake may be sufficient to achieve the maximal reduction in MetS odds.

### 3.2. Total Daily Consumption of Dairy Foods and Metabolic Syndrome

Table 2 presents the main characteristics of the studies that evaluated the association between dairy products consumption and metabolic syndrome in adults [37,38,39,40,41,42,43,44,45,46,47]. Overall, the included studies comprised a total of 70,985 men and 129,952 women. With regard to study location, seven studies were conducted in Asia, two in Europe and in Brasil and one in the USA. Dietary calcium intake was assessed using FFQs and/or 24–48 h dietary recalls. Study quality was rated as high in nine studies, moderate in two, and low in one.

**Figure 4:**
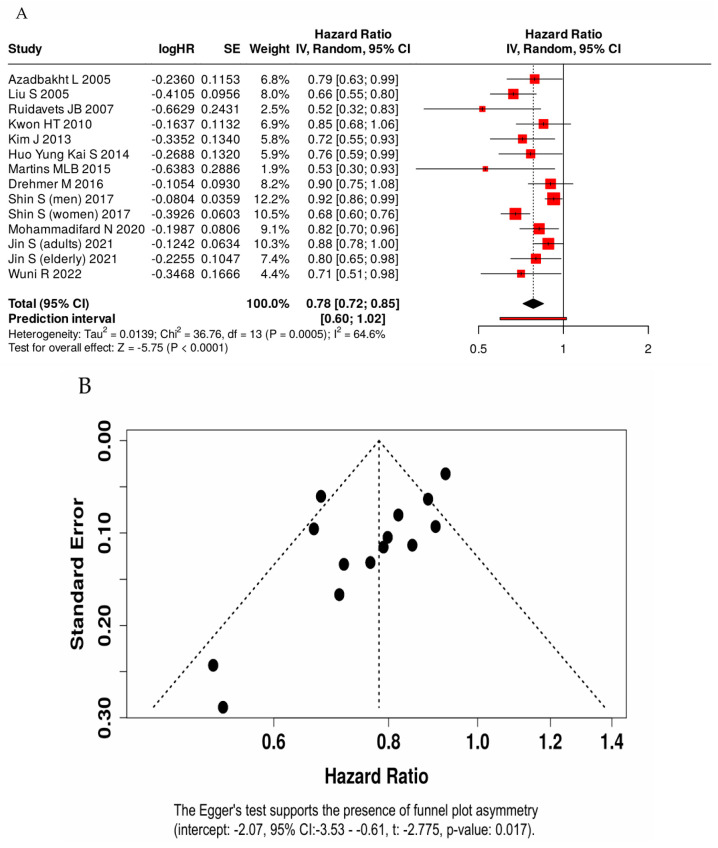
Forest plot (**A**) and funnel plot (**B**) of the association between the highest versus lowest category of dairy food/product consumption and metabolic syndrome in adults [27,37,38,39,40,41,42,43,44,45,46,47].

Using a random-effects model, higher total calcium intake was associated with lower odds of metabolic syndrome, with a pooled odds ratio of 0.78 (95% CI: 0.72–0.85) for the highest compared with the lowest intake category. Thus, the highest level of dairy products consumption, compared with the lowest, was associated with a 22% reduced odds of metabolic syndrome [Figure 4]. Moderate-to-substantial heterogeneity was observed across studies (I^2^ = 64.6%). The estimated between-study variance was modest (τ^2^ = 0.013; τ ≈ 0.11), indicating limited absolute variability in effect sizes across studies. Egger’s regression test showed significant funnel plot asymmetry (*p* = 0.016), which was confirmed by Begg rank correlation test (Kendall’s τ = −0.56, *p* = 0.005). Although both Egger’s and Begg’s tests indicated the presence of small-study effects, the trim-and-fill analysis showed an attenuation of the pooled effect after imputing potentially missing studies, while the association remained statistically significant. This suggests that publication bias may have inflated the magnitude of the effect, but it is unlikely to fully account for the observed association.

**Figure 5:**
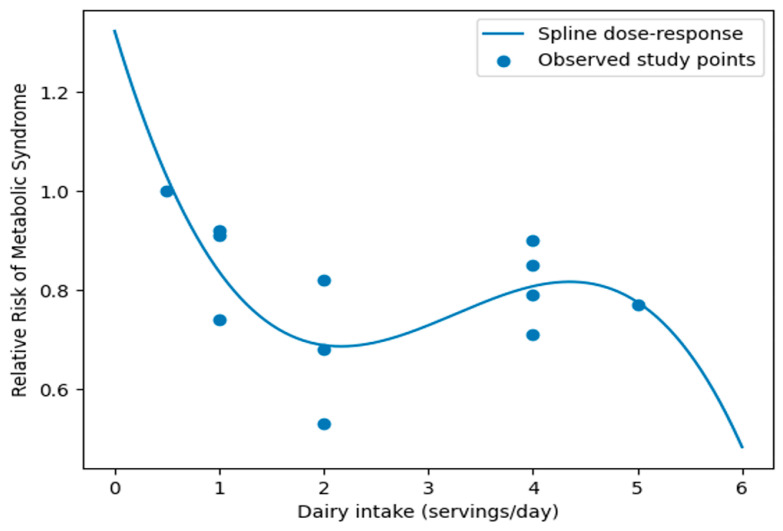
Nonlinear dose–response spline: dairy products and metabolic syndrome.

We conducted additional meta-regression analyses to explore potential sources of heterogeneity. The models included geographic region, sex group, and dietary assessment method as study-level moderators. The results indicated that sex significantly moderated the association between dairy products and metabolic syndrome, with a stronger inverse association observed in women compared with men (β = −0.302, *p* = 0.003). In contrast, geographic region and dietary assessment method were not significantly associated with the effect size. These findings suggest that sex differences may partly explain the between-study heterogeneity observed in the analysis (Table S3B of the Supplementary Material). Dose–response relationships were examined in nine studies where dairy product intake could be evaluated in terms of servings per day, using a two-stage random-effects dose–response meta-analysis based on the Greenland and Longnecker method. A linear dose–response analysis showed that 1-serving/day increment in dairy intake was associated with an 8% lower risk of metabolic syndrome (OR 0.92, 95% CI 0.90–0.95). A restricted cubic spline dose–response meta-analysis showed a nonlinear inverse association between dairy intake and metabolic syndrome. The steepest reduction in risk was observed up to approximately 2 servings/day, after which the association plateaued [Figure 5].

**Figure 6:**
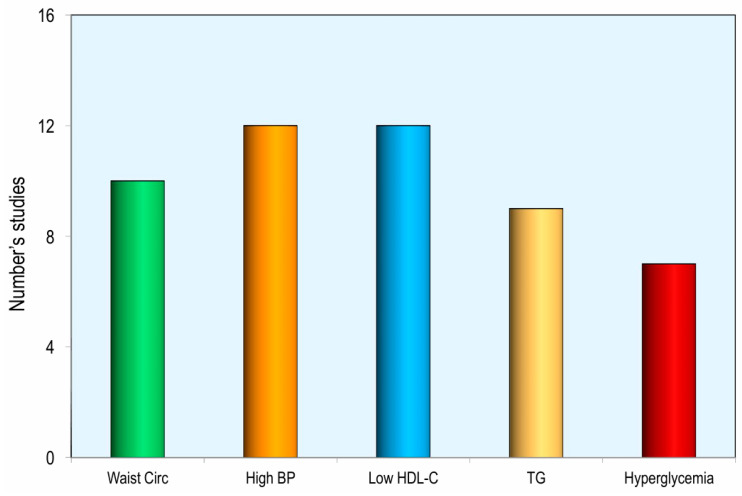
Number of studies reporting a significant difference in the odds of individual metabolic syndrome components between the highest and lowest calcium intake groups (BP = Blood Pressure; C-HDL = High-Density Lipoprotein Cholesterol, TG = Triglycerides).

Sixteen studies examined the association between dietary calcium intake and the odds of individual components of metabolic syndrome. Higher calcium intake, from either total dietary sources or dairy products, was associated with significantly lower odds of elevated blood pressure and low HDL cholesterol in 12 studies, increased waist circumference in 10 studies, elevated triglycerides in 9 studies, and fasting hyperglycemia in 7 studies [Figure 6].

## 4. Discussion

The majority of cross-sectional studies included in this meta-analysis indicate that dietary calcium intake is inversely associated with the odds of metabolic syndrome in adults. This association was not observed only in the studies by Bruscato and Motamed [29,31]. In particular, in the study by Bruscato et al., the lack of association may be explained by the characteristics of the study population, which consisted exclusively of postmenopausal women older than 65 years, suggesting that nutritional factors may exert a stronger influence on MetS development at younger ages [29]. Moreover, estrogen deficiency may contribute to these age-related differences, as estrogens enhance calcium absorption and exert beneficial metabolic effects, including improvements in insulin sensitivity, reductions in inflammation, and limitation of abdominal fat accumulation [48,49]. Supporting this evidence, studies that analyzed men and women separately consistently reported a stronger protective effect of calcium intake in women than in men [28,30,32,33,35]. Similarly, in the study by Shin S. et al. [44], higher milk consumption was associated with lower odds of MetS, with a more pronounced inverse association in females. Numerous studies have demonstrated an association between dairy products with MetS [37,50]. However, this association often loses statistical significance after adjustment for calcium intake, highlighting the central role of this mineral [42]. Milk is a rich source of bioactive compounds that may improve metabolic efficiency and target multiple components of MetS; nevertheless, calcium appears to represent a key mediator of these beneficial effects. Importantly, the source of calcium seems to be a critical determinant. In a study by Shin BR et al., a paradoxical finding was reported, whereby obese males with the highest calcium intake who did not consume dairy products showed an increased incidence of MetS [34]. This observation suggests that both the dietary source of calcium and obesity status may significantly modify the association between calcium intake and MetS risk. This factor should therefore be carefully considered when interpreting associations between calcium intake and metabolic outcomes. Whether calcium supplementation can replicate the beneficial effects observed with dietary calcium remains unclear, and the optimal supplemental dose has yet to be established. Moreover, although higher dietary calcium intake has been associated with a reduced risk of metabolic syndrome in several studies, elevated serum calcium concentrations have conversely been linked to an increased likelihood of developing metabolic syndrome [51]. This apparent discrepancy suggests that the metabolic effects of calcium may depend on its dietary source and physiological regulation rather than on total systemic calcium levels alone. Collectively, these findings underscore the importance of promoting an overall healthy dietary pattern rather than reliance on isolated nutrient supplementation. Consistent with the previous literature, the present meta-analysis confirms that higher total dietary calcium intake and dairy consumption are inversely associated with the risk of individual components of metabolic syndrome [13,15]. Furthermore, the protective effect of dietary calcium appears to be more pronounced for blood pressure and lipid profiles. Several studies have reported that dairy foods may exert beneficial effects on blood pressure [17]. The beneficial effects of dairy foods on metabolic syndrome may extend beyond their calcium content and may be attributed to the complex dairy matrix, likely reflecting the combined action of bioactive peptides, whey proteins, specific fatty acids, vitamins, and fermentation-derived compounds that can favorably modulate insulin sensitivity, blood pressure, inflammation, and gut microbiota composition [52]. In particular, adequate calcium intake has been shown to modulate the renin–angiotensin–aldosterone system and improve the sodium–potassium balance, thereby contributing to blood pressure reduction [53]. Blood pressure is tightly regulated by intracellular calcium concentrations in vascular smooth muscle cells, which influence vasoconstriction and vascular tone. Low dietary calcium intake appears to activate multiple hypertensive mechanisms, including increased secretion of the parathyroid hormone, which elevates intracellular calcium directly and via calcitriol activation, and stimulation of the renin–angiotensin–aldosterone system, leading to enhanced sodium and water reabsorption and subsequent expansion of vascular volume [54]. Increased dietary calcium reduces intracellular Ca^2+^ influx in adipocytes, thereby decreasing fatty acid synthesis and enhancing lipolysis, which results in reduced triglyceride storage. Moreover, calcium, which is abundant in milk and yogurt, can bind fatty acids and bile acids in the intestine, increasing fecal fat excretion and inhibiting lipid reabsorption [16]. Consequently, higher calcium intake may contribute to an improvement in the HDL-cholesterol to LDL-cholesterol ratio [16,18] and a reduction in fat mass, particularly in females [55]. However, despite the aforementioned positive effects of dietary calcium intake on the various components of the metabolic syndrome, studies that have evaluated the relationship between calcium intake and cardiovascular diseases such as stroke and coronary syndromes have reported inconclusive results [56]. Beyond lipid metabolism, calcium may exert additional benefits through its effects on oxidative stress and inflammation. Both oxidative stress and chronic low-grade inflammation are recognized as key contributors to the development and progression of metabolic syndrome [57]. Evidence suggests that adherence to a Mediterranean dietary pattern and higher dietary calcium intake may exert antioxidant effects, providing an additional layer of protection against MetS [58]. From a nutrigenomic perspective, the role of calcium appears particularly relevant. Nutrigenomics, which investigates interactions between nutrients and genetic variants, has shown that dietary patterns, such as high-fat diets, can modulate genetic susceptibility to MetS [59]. In a study conducted in a Korean population, Kim et al. reported that higher calcium intake attenuated the risk of MetS among carriers of the minor alleles of rs6445834 in the ARHGEF3 gene, rs10850335 in the TBX5 gene, and rs180349 in the BUD13 gene [30]. These findings suggest that calcium intake may interact with specific genetic profiles to influence metabolic health outcomes, highlighting its potential role in personalized nutrition strategies.

Several limitations of this study should be acknowledged. First, all included studies were cross-sectional, which precludes causal inference and allows for potential residual confounding and reverse causation. Second, moderate-to-substantial heterogeneity was observed, likely reflecting differences in study populations, dietary assessment methods, and confounder adjustment. Third, calcium intake was primarily assessed using self-reported dietary instruments, which are inherently prone to measurement error. Fourth, evidence of small-study effects was detected in the analysis of dairy consumption, which may have led to an overestimation of effect sizes. Fifth, since the study did not report results based on identical covariate models, standardizing the adjustment across studies was not feasible. Finally, the predominance of studies conducted in Asian and Western populations may limit the generalizability of the findings to other ethnic and geographic groups. This systematic review and meta-analysis also has several notable strengths. First, it is based on a large overall sample size, providing high statistical power and precise effect estimates. The inclusion of studies assessing both total dietary calcium intake and calcium derived from dairy products allowed evaluation of different dietary sources of calcium. Most included studies were of moderate-to-high methodological quality and involved apparently healthy adults, reducing the likelihood of disease-related reverse causation. The consistency of inverse associations across several components of metabolic syndrome, particularly blood pressure and HDL cholesterol, supports the biological plausibility of the findings. Additionally, the use of random-effects models and multiple approaches to assess publication bias enhanced the robustness and generalizability of the results.

## 5. Conclusions

In conclusion, this systematic review and meta-analysis provides robust evidence of an inverse association between dietary calcium intake and metabolic syndrome in adults, with a more pronounced protective effect in women. Evidence suggests that calcium is a key mediator of the beneficial effects of dairy products on metabolic health, particularly on blood pressure and lipid profiles. Biological plausibility is supported by mechanisms involving lipid metabolism, blood pressure regulation, inflammation, and gene–diet interactions. However, the cross-sectional nature of the included studies precludes any inference of causality between calcium intake and MetS. Well-designed prospective and interventional studies are warranted to clarify whether this relationship is causal and to define optimal dietary recommendations. Overall, these findings support recommendations promoting adequate calcium intake from foods within healthy dietary patterns, given its potential contribution to a lower risk of metabolic syndrome and better cardiometabolic health.

## Figures and Tables

**Table 1:** Main characteristics of the eligible cross-sectional studies included in the systematic review and meta-analysis investigating the association between total dietary calcium intake and metabolic syndrome in adults.

Author/Year/Country	MetS Assessment	Dietary Assessment	Study Population	Consumption Amount	OR/RR (95% CI)	Conclusions
Liu S (2005) USA [27]	NCEP-ATPIII	FFQ and 1 W dietary record	Healthy ♀ 10,066 Age > 45 years	Dietary calcium (mg/day) Q1: 223–561 Q2: 562–683 Q3: 684–805 Q4: 806–1004 Q5: 1005–2596	1 0.89 (0.75–1.06) 0.87 (0.73–1.04) 0.81 (0.67–0.98) 0.74 (0.60–0.92)	Lower prevalence of MetS in women with higher dietary calcium intake.
Cho JC (2009) Korea [28]	NCEP-ATPIII	24 h dietary recall	Healthy ♂ 4118 ♀ 3359 PreM ♀ 1864 PostM Age > 20 years	Dietary calcium (mg/day) Men Q1: <347; Q2: 347–510 Q3: 511–736 Q4: >736. PreM Women Q1: <300 Q2: 300–442 Q3: 443–630 Q4: >530 PostM Woman Q1: 253 Q2: 253–396 Q3: 397–608 Q4: >608	1 0.97 (0.78–1.24) 0.93 (0.76–1.22) 0.79 (0.61–1.03) 1 1.11 (0.78–1.57) 1.06 (0.74–1.53) 0.98 (0.65–1.45) 1 0.93 (0.69–1.27) 0.91 (0.66–1.26) 0.64 (0.45–0.90)	In PreM women, increased calcium intake is related to a decreased risk of heavy MetS.
Bruscato NM (2010) Brazil [29]	IDF	24 h dietary recall	Healthy ♀ 284 MetS = ♀ 91 No MetS = ♀ 193 aged ≥ 60 years	Median Dietary calcium (mg/day) Q1: 669 Q2: 756 Q3: 789 Q4: 748	1 2.43 (1.12–5.27) 1.59 (0.73–3.40) 1.50 (0.68–3.31)	Dietary factors do not influence the risk of MetS in elderly women.
Kim K (2012) Korea [30]	ATPIII	FFQ	Healthy ♂ 3846 ♀ 4185 Aged 39–70 years	Dietary calcium (mg/day) Men T1: 69.0–345.4 T2: 345.5–473.0 T3: 473.1–1386 Women T1: 43.4–360.8 T2: 360.9–516.1 T3: 516.2–1715.1	1 0.92 (0.76–1.13) 0.77 (0.61–0.98) 1 0.81 (0.67–0.98) 0.65 (0.52–0.81)	Higher calcium intake was inversely associated with MetS risk in both men and women.
Motamed S (2013) Iran [31]	IDF	24 h dietary recall	Healthy ♂ 1231 ♀ 2399 Aged 35–65 years	Median Dietary calcium (mg/day) Q1: 430.5 Q2: 603.3 Q3: 736.8 Q4: 887.9 Q5: 1130.1	1 1.04 (0.8–1.2) 1.08 (0.8–1.2) 1.12 (0.9–1.4) 1.17 (0.9–1.4)	No association between dietary calcium intake and MetS.
Al-Daghri NM (2013) Saudi Arabia [19]	IDF	Two 24 h dietary recall	Healthy ♂ 87 ♀ 98 Aged 19–60 years	Median Dietary calcium (mg/day) Q1: 340.0 Q2: 506.7 Q3: 669.6 Q4: 1003.6	6.1 (1.9–19.5) 1.3 (0.42–3.8) 1.3 (0.46–3.7) 1	In women, there was an inverse association of calcium intake and prevalence of MetS.
Moore-Schiltz L (2015) USA [32]	NCEP/ATP III	24 h dietary recall	Healthy MetS (−) ♂ 2827 ♀ 2742 Age 45.8 years MetS (+) ♂1722 ♀1857 Aged 57.3 years	Dietary Calcium Intake (mg/day) All Q1: <547 Q2: 547–815 Q3: 816–1172 Q4: >1172 Men Q1: <572 Q2: 572–860 Q3: 861–1224 Q4: >1224 Woman Q1: <524 Q2: 524–773 Q3: 774–1109 Q4: >1109	1 1.03 (0.89–1.17) 1.01 (0.77–1.10) 0.92 (0.82–1.10) 1 1.06 (0.86–1.30) 1.15 (0.93–1.42) 1.03 (0.78–1.36) 1 0.89 (0.73–1.09) 0.88 (0.74–2.06) 0.73 (0.59–0.89)	Women who met RDA for calcium (1000–2000 mg/die) had reduced odds of MetS.
Shin SK (2015) Korea [33]	NCEP-ATPIII	FFQ	Healthy ♂ 2491 ♀ 3884 Aged ≥ 40 years	Calcium Intake (mg/day) Men Q1: 37.5–238.8 Q2: 238.8–322.6 Q3: 322.6–435.2 Q4: 435.5–1371 Women Q1: 32.5–211.6 Q2: 211.7–302.1 Q3: 302.0–416.2 Q4: 416.2–1150.2	Men 1 0.98 (0.78–1.18) 0.86 (0.65–1.14) 0.82 (0.60–1.14) Women 1 0.96 (0.78–1.18) 0.85 (0.67–1.06) 0.71 (0.54–0.94)	Calcium intake was inversely related to MetS risk in women but not in men.
Shin BR (2016) Korea [34]	NCEP-ATPIII	FFQ and 24 h dietary recall	Healthy normal Weight ♂ 3889 Age 44.7 ± 0.36 years Obese ♂ 2057 Age 43.9 ± 0.3 years	Dietary calcium intake (mg/day) Q1: 30.9–333.8 Q2: 333.9–493.0 Q3: 493.1–716.6 Q4: 716.7–5049.1 Q1: 30.9–333.8 Q2: 333.9–493.0 Q3: 493.1–716.6 Q4: 716.7–5049.1	Normal weight 1 0.90 (0.66–1.26) 0.83 (0.58–1.18) 0.86 (0.57–1.29) Obese 1 1.44(1.00–2.06) 1.27 (0.88–1.81) 1.61 (1.12–2.36)	Obese males with higher calcium intake but not dietary product intake had higher incidence of MetS.
Pannu PK (2017) Australia [20]	IDF	24 h dietary recall	Healthy MetS (−) ♂ 1237 ♀ 1565 Age 41 years MetS (+) ♂ 344 ♀ 257 Age 52 years	Dietary calcium intake (mg/day) T1: 579 T2: 858 T3: 1233	1 0.73 (0.56–0.96) 0.63 (0.46–0.86)	Adults with MetS had lower calcium intake.
Kim MK (2017) Korea [35]	NCEP-ATPIII	24 h dietary recall	Healthy ♂ 5953 Age 49.40 ± 15.98 years ♀ 4258 PreM Age 35.86 ± 8.26 years ♀ 4494 PostM Age 62.63 ± 9.86 years	Dietary calcium (mg/day) Men Q1: ≤400 Q2: 401–800 Q3: 801–1200 Q4: >1200 PreM Women Q1: ≤400 Q2: 401–800 Q3: 801–1200 Q4: >1200 PostM Woman Q1: ≤400 Q2: 401–800 Q3: 801–1200 Q4: >1200	Men 1 1.01 (0.81–1.25) 0.90 (0.65–1.26) 1.94 (1.23–3.05) PreM Women 1 0.79 (0.55–1.12) 1.17 (0.64–2.13) 0.72 (0.23–2.24) PostM Women 1 0.78 (0.64–0.96) 0.92 (0.63–1.33) 0.80 (0.41–1.55)	Excessive calcium intake increased the prevalence of MetS in men.
Choi B (2024) Korea [36]	NCEP-ATPIII	FFQ	Healthy MetS (−) ♂ 31,210 ♀ 65,545 MetS (+) ♂ 12,640 ♀ 21,028 Age 40–70 years	Dietary calcium intake (mg/day) Q1 Q2 Q3 Q4	Men MetS (+) 0.94 (0.92–0.97) Women MetS (+) 0.92 (0.90–0.94)	The total calcium intake was lower in subjects with MetS (+).

**Abbrevations:** NCEP ATP III, the Third Report of the National Cholesterol Education Program Adult Treatment Panel; FFQ, food frequency questionnaires; W, week; ♀, female; Q, quartile/quintile; MetS, Metabolic Syndrome, ♂, male; preM, premenopausal; postM, postmenopausal; IDF, the International Diabetes Federation definition; RDA, Recommended Daily Allowance.

**Table 2:** Main characteristics of the eligible cross-sectional studies included in the systematic review and meta-analysis on the association between dairy foods/products consumption and metabolic syndrome in the adult population.

Author/Year/Country	MetS Assessment	Dietary Assessment	Study Population	Consumption Amount	OR/RR (95% CI)	Conclusions
Azadbakht L (2005) Ira [37]	NCEP-ATPIII	FFQ and 24 h dietary recall	Healthy ♂ 357 ♀ 470 Age 18–74 years	Serving/day Q1: <1.7 Q2: 1.7–2.3 Q3: 2.3–3.1 Q4: >3.1	1 0.96 (0.69–1.20) 0.92 (0.84–1.12) 0.82 (0.63–0.99)	Highest Q of diary Ca consumption had lower OR of having MetS.
Liu S (2005) USA [27]	NCEP-ATPIII	FFQ and 1 W dietary record	Healthy women ♀ 10,066 Age > 45 years	Serving/day Q1: <0.91 Q2: 0.91–1.41 Q3: 1.42–1.99 Q4: 2.00–3.00 Q5: >3.00	1 0.85 (0.71–1.00) 0.76 (0.64–0.91) 0.77 (0.64–0.92) 0.66 (0.55–0.80)	Increased consumption of dairy product is associated with lower prevalence of MetS.
Ruidavets JB (2007) France [38]	NCEP-ATPIII	3 d dietary record	Healthy men ♂ 912 Age 45–64 years	Dairy product (g/day) Q1: 32.6 Q2: 25.0 Q3: 21.8 Q4: 18.8 Q5: 19.9	1 0.69 (0.44–1.09) 0.58 (0.36–0.92) 0.46 (0.29–0.75) 0.51 (0.32–0.83)	High consumption of dairy products is associated with decreased odds of having MetS.
Kwon HT (2010) Korea [39]	NCEP-ATPIII	FFQ and 24 h dietary recall	Healthy ♂ 2052 ♀ 2838 Age ≥ 19 years BMI > 23 Kg/m^2^	Frequency of milk intake Q1: rarely Q2: ≤1/week Q3: 2–6/week Q4: ≥1 day	1 0.86 (0.69–1.07) 1.11 (0.68–1.06) 0.85 (0.68–1.06) OR = 0.72 (0.587–0.92)	Increased frequency of milk had an inverse relationship with MetS among overweight adults.
Kim J (2013) Korea [40]	JIS	FFQ and 24 h dietary recall	Healthy ♂ 1993 ♀ 2869 Age ≥ 19 years	Yogurt Q1: None or rarely Q2: ≤2–3 per month Q3: ≤4–6 per week Q4: ≥once day Milk Q1: None or rarely Q2: ≤2–3 per month Q3: ≤4–6 per week Q4: ≥once day	1 0.88 (0.73–1.06) 0.77 (0.62–0.95) 0.71 (0.48–1.05) 1 1.12 (0.91–1.39) 0.89 (0.73–1.10) 0.71 (0.55–0.93)	Milk and yogurt consumption are inversely associated with the risk of the MetS.
Huo Yung Kai S (2014) France [41]	ATPIII	3 d dietary record	Healthy ♂ 1550 ♀ 1428 Age 35–74 years	Total dairy intake (g/1000 kJ) Q1: 0–13.2 Q2: 13.3–23.1 Q3: 23.2–36.3 Q4: 36.4–266.0	1 0.97 (0.76–1.24) 0.92 (0.72–1.09) 0.76 (0.59–0.99)	High-fat dairy intake does not reduce MetS risk.
Martins MLB (2015) Brasil [42]	IDF	FFQ	Healthy ♂ 984 ♀ 1047 Age 23–25 years	Portions/day Q1: 0.0–0.6 Q2: 0.7–1.2 Q3: 1.3–1.7 Q4: 1.8–2.6 Q5: 2.7–14.2	1 0.94 (0.61–1.43) 1.06 (0.68–1.65) 0.79 (0.49–1.26) 0.53 (0.30–0.93)	The association between dairy consumption and MetS lost significance after adjustment for calcium intake.
Drehmer M (2016) Brasil [43]	JIS	FFQ	Healthy ♂ 4445 ♀ 5390 Age 35–74 years	Dairy serving/day Q1: <1 Q2: 1–2 Q3: >2–4 Q4: >4	1 0.95 (0.80–1.13) 0.91 (0.77–1.07) 0.90 (0.75–1.08)	A graded inverse association for MetScore with total dairy and full-fat dairy but not with low-fat dairy intake. Associations were no longer present after additional adjustments for dairy-derived saturated fatty acids.
Shin S (2017) Korea [44]	NCEP-ATPIII	FFQ	Healthy ♂ 43,786 ♀ 86,738 Age 40–69 years	Milk Consumption Men Q1: Non or rarely Q2: ≤2/week Q3: 3–6/week Q4: ≥1 day Women Q1: Non or rarely Q2: ≤2/week Q3: 3–6/week Q4: 1 day Q5: ≥2 day	Men 1 0.94 (0.89–1.00) 0.93 (0.86–1.00) 0.92 (0.86–0.99) Women 1 0.88 (0.84–0.92) 0.86 (0.82–0.91) 0.85 (0.81–0.90) 0.68 (0.60–0.76)	Higher milk consumption associated with the lower odds of MetS.
Mohammadifard N (2020) Iran [45]	ATPIII	FFQ	Healthy ♂ 4785 ♀ 4768 Aged 19 years	Total diary intake/W Q1: <12 Q2: 12–16 Q3: 17–21 Q4: >21	1 0.88 (0.73–1.08) 0.85 (0.65–0.98) 0.84 (0.70–0.96)	Total diary intake is inversely associated with MetS.
Jin S (2021) Korea [46]	NCEP-ATPIII	24 h dietary recall	Healthy Adults ♂ 7488 ♀ 10,718 Age 19–64 years Healthy Elderly ♂ 2200 ♀ 2913 Age > 65 years	Serving/day T1: 0 T2: <1 T3: ≥1 T1: 0 T2: <1 T3: ≥1	Adults 1 1.01 (0.85–1.20) 0.91 (0.78–1.06) Elderly 1 0.95 (0.73–1.24) 0.74 (0.57–0.96)	One or more servings of dairy products were associated with lower MetS in adult men and in elderly women.
Wuni R (2022) India [47]	IDF	FFQ	Healthy Adults ♂ 433 ♀ 600 Age 36 (IQR15) years	Total Dairy Products (g/day) Lowest: 208 (116)–1.4 cups Medium: 411(144)–3 cups High: 755 (228)–5 cups	1 0.87 (0.69–1.09) 0.71 (0.51–0.98)	Increasing the consumption of dairy might help to reduce MetS.

**Abbrevations:** NCEP ATP III, the Third Report of the National Cholesterol Education Program Adult Treatment Panel; FFQ, food frequency questionnaires; ♂, male; ♀, female; Q, quartile; OR, odds ratio; MetS, Metabolic Syndrome; BMI, Body Mass Index; IDF, the International Diabetes Federation definition; W, week; T, tertiles.

## Data Availability

Where no new data were created.

## Supplementary Materials

The following supporting information can be downloaded at: https://www.mdpi.com/article/10.3390/nu18061006/s1, Supplementary Table S1A: Details of quality assessment of included studies in the systematic review and met-analysis based on Newcastle-Ottawa Scale adapted for cross-sectional studies on dietary calcium intake. Supplementary Table S1B: Details of quality assessment of included studies in the systematic review and met-analysis based on Newcastle-Ottawa Scale adapted for cross-sectional studies on dairy products. Supplementary Table S2A: GRADE evidence Profile of dietary calcium intake and MetS. Supplementary Table S2B: GRADE evidence Profile of dairy products and MetS. Supplementary Table S3A: Meta-regression analysis Dietary Calcium Intake and MetS. Supplementary Table S3B: Meta-regression analysis Dairy Products and MetS. References [19,20,27,28,29,30,31,32,33,34,35,36,37,38,39,40,41,42,43,44,45,46,47] are cited in the supplementary materials.

## Abbreviations

The following abbreviations are used in this manuscript:

MetS	Metabolic syndrome
ORs	Odds Ratios
CIs	Confidence intervals
FFQs	Food Frequency Questionnaires
LDL	Low-Density Lipoprotein
HDL	High-Density Lipoprotein
DASH	Dietary Approaches to Stop Hypertension
NOS	Newcastle-Ottawa Scale
NCEP ATPIII	Third Report of the National Cholesterol Education Program Adult Treatment Panel
IDF	International Diabetes Federation
JIS	Joint Interim Statement of the International Diabetes Federation
