# Supplementary material for: Association Between Dietary Calcium or Dairy Product Intake and Metabolic Syndrome Risk: A Systematic Review and Meta-Analysis
Source: Nutrients. 2026 Mar 22;18(6):1006. doi: 10.3390/nu18061006 (PMC13029373; doi:10.3390/nu18061006)
Supplement: Supplementary file 1 [file nutrients-18-01006-s001.zip › nutrients-4181642-supplementary.pdf]

Supplemetary Table 1 A: Details of quality assessment of included studies in the systematic review and met-analysis based on Newcastle-Ottawa Scale adapted for cross-sectional studies on dietary calcium intake.

	Representativeness of the sample	Sample size	Non responders described	Validated Measurement Tool	Control for confounders	Validated Outcome Assesement	Appropriate Statistical Tests	Precision of estimates (p value)	Total Score
**Liu S 2005[27]**		*	*	*	*	*	*	*	7
**Cho JC 2009[28]**	*	*	*	*	**	*	*	*	9
**Bruscato NM 2010[29]**	*		*		*		*		4
**Kim K 2012[30]**	*	*	*	*	*	*	*	*	8
**Motamed S 2013[31]**		*	*	*	*	*	*		6
**Al-Daghri NM 2013[19]**	*		*	*	*	*	*	*	7
**Moore-Shiltz L 2015[32]**		*	*	*	*	*	*	*	7
**Pannu PK 2017[20]**	*	*	*	*	*	*	*		7
**Shin S 2015[33]**	*	*	*	*	*	*	*	*	8
**Shin BR 2016[34]**	*	*	*	*	*	*	*	*	8
**Kim MK 2017[35]**	*	*	*		*	*	*		6
**Choi B 2024[36]**	*	*	*		*	*	*		6

Supplemetary Table 1 B: Details of quality assessment of included studies in the systematic review and met-analysis based on Newcastle-Ottawa Scale adapted for cross-sectional studies on dairy produts.

	**Representativeness of the sample**	**Sample size**	**Non responders described**	**Validated Measurement Tool**	**Control for confounders**	**Validated Outcome Assesement**	**Appropriate Statistical Tests**	**Precision of estimates (p value)**	**Total Score**
**Azadbakht L 2005[37]**	*		*	*	*	*	*	*	7
**Liu S 2005[27]**	*	*		*	*	*	*	*	7
**Ruidavets JB 2007[38]**	*	*			*	*			4
**Kwon HT 2010[39]**	*	*	*	*	*	*	*		7
**Kim J 2013[40]**	*	*	*	*	*	*	*	*	8
**Huo Yung S 2014[41]**	*	*	*	*	*	*	*	*	8
**Martins MLB 2015[42]**	*	*	*		*	*	*	*	7
**Drehmer M 2016[43]**	*	*	*	*	*	*	*		7
**Shin S 2017[44]**	*	*	*	*	*	*	*	*	8
**Mohammadifard N 2020[45]**	*	*		*	*	*	*		6
**Jin S 2021[46]**	*	*	*	*	*	*	*	*	8
**Wuni R 2022[47]**	*		*	*	*	*	*		6

TABLE - 2A

N° studies	Study design	Risk of bias	Inconsistency	Pubblication bias	Imprecision	Other consideration	Effect Relative (95%C.I.)	Certainty
12 articles 20 effect sizes	Observational studies (cross-sectional)	Not serious (a)	Serious (b)	Not serius (c)	Not serius	Large population dose responce (d)	HR 0.85 (C.I. 0.80-0.91)	●●●○ Moderate

(a) quality assessment was evaluated on Newcastle-Ottawa scale and was considered adequate. The cross-sectional nature of studies limit causal inference

(b) substantial heterogeneity (I^2= 70.1%) was observed; although below the threshold of 75% used to define high heterogeneity

(c) Funnel-plot did not reveal substantial asymmetry and Eger's test was not significant

(d) The presence of a clear dose-response supports up grading the certainty of evidence

Supplementary Table 2 A : GRADE evidence Profile of dietary calcium intake and MetS

TABLE - 2B

N° studies	Study design	Risk of bias	Inconsistency	Pubblication bias	Imprecision	Other consideration	Effect Relative (95%C.I.)	Certainty
12 articles 14 effect sizes	Observational studies (cross-sectional and color studies)	Not serious (a)	Serious (b)	Serius (c)	Not serius	Large population dose responce (d)	HR 0.78 (C.I. 0.60-1.02)	●●○○ Low

(a) quality assessment was evaluated on Newcastle-Ottawa scale and was considered adequate. The cross-sectional nature of studies limit causal inference

(b) substantial heterogeneity (I^2= 64.6%) was observed; although below the threshold of 75% used to define high heterogeneity

(c) Funnel-plot did not reveal elevated asymmetry but Eger's test was significant

(d) The presence of a clear dose-response supports up grading the certainty of evidence

Supplementary Table 2 B : GRADE evidence Profile of dairy products and MetS

Moderator	β (SE)	p-value	95% CI
Middle East	0.105 (0.618)	0.865	−1.107 to 1.317
North America	−0.045 (0.166)	0.786	−0.369 to 0.279
Oceania	−0.619 (0.354)	0.080	−1.312 to 0.074
South America	0.626 (0.475)	0.187	−0.304 to 1.556
**Women (vs men)**	**−0.275 (0.133)**	**0.038**	**−0.534 to −0.015**
FFQ (vs 24-h recall)	−0.067 (0.138)	0.626	−0.338 to 0.203

Supplementary Table 3A: Meta-regression analysis Dietary Calcium Intake and MetS

Moderator	β (SE)	p-value	95% CI
**Intercept**	-0.083 (0.064)	0.190	-0.208 to 0.041
**Continent**			
North America	0.147 (0.183)	0.423	-0.212 to 0.506
South America	0.031 (0.139)	0.823	-0.241 to 0.304
**Sex group**			
Mixed	-0.115 (0.115)	0.318	-0.341 to 0.111
Women	**-0.302 (0.102)**	**0.003**	**-0.502 to -0.102**
**Dietary assessment method**			
FFQ + recall	-0.023 (0.126)	0.858	-0.270 to 0.225
Recall	0.042 (0.124)	0.738	-0.202 to 0.285

Supplementary Table 3B: Meta-regression analysis Diary Products and MetS
